# Schizophrenia in DiGeorge Syndrome: A Unique Case Report

**DOI:** 10.7759/cureus.3142

**Published:** 2018-08-14

**Authors:** Sukaina Rizvi, Ali M Khan, Hina Saeed, Akeem M Aribara, Alexis Carrington, Alexa Griffiths, Abdul Mohit

**Affiliations:** 1 Psychiatry, Kings County Hospital Center, Brooklyn, USA; 2 Psychiatry Resident, University of Texas Rio Grande Valley, Harlingen, Texas, USA; 3 Psychiatry, Sindh Medical, Ontario , CAN; 4 Psychiatry, Kings County, Brooklyn , USA; 5 Psychiatry, St. George's University School of Medicine, Brooklyn, USA; 6 Others, St. George, Brooklyn, USA; 7 Behavioral Health, Kings County Hospital Center, New York, USA

**Keywords:** psychosis, basal ganglia calcification, digeorge syndrome, schizophrenia

## Abstract

Herein we present the unique case of a 21-year-old African American woman who presented with psychotic features and the incidental finding of basal ganglia calcifications on computed tomography (CT) scan of the head. She was initially presumed to have Fahr’s syndrome in the context of idiopathic bilateral basal ganglia calcifications and psychotic features. Genetic testing performed revealed the deletion of 22q11.2, thus establishing the diagnosis of DiGeorge syndrome. This case highlights the importance of noticing subtle physical exam findings along with laboratory findings as this led to the diagnosis of DiGeorge syndrome for this patient. This case is unique in two aspects; first, the finding of basal ganglia calcification via CT of the brain in patients with DiGeorge syndrome has rarely been reported in the literature. Second, this case highlights the strong genetic predisposition for schizophrenia in patients with DiGeorge syndrome.

## Introduction

This case report serves to illustrate the occurrence of psychotic disturbances in patients with DiGeorge syndrome. In accordance with the recent data, 25% to 33% of individuals with DiGeorge syndrome develop psychiatric features [1]. In comparison to other cases that have been published, this DiGeorge syndrome case is unique as the first break psychosis was the initial presentation of the 22q11.2 deletion. This highlights the variable clinical presentation of DiGeorge syndrome in each patient and the need for a multidisciplinary methodology to diagnose and treat it.

## Case presentation

The patient is a 21-year-old African American woman, unemployed, living in Brooklyn, New York, with no prior psychiatric history. She was brought in by emergency medical services at the request of her mother due to the patient’s increased aggression and paranoia at home. As per her mother’s account, the patient began acting bizarre two months ago when she left her home to stay with her boyfriend. The mother was contacted by the patient a few days later concerning paranoid ideation that people were trying to kill her. The patient also tried to attack her mother while she was driving a car. Additionally, she also started accusing her family members of being replacements (Capgras syndrome). As per her mother’s account, the patient had been intrusive towards strangers on the street, reading the Bible and getting in people’s faces.

When the patient was presented to the hospital, she seemed very confused, internally preoccupied, disorganized, and providing delayed and inappropriate responses to questions when asked. This was her first psychiatric presentation. It was noted that she had an elongated face with small ears rotated backward and exhibited hypernasal speech. She appeared to be labile, crying for no apparent reason then later singing out loud. The patient refused to come out of her room for an initial interview. During her interview, she remained evasive and guarded. Her thought process was illogical, and her thought content was delusional (e.g., she thought her mother was the devil). She denied visual, tactile, olfactory, and gustatory hallucinations but endorsed hearing Jesus’s voice telling her that everything was going to be ok. She denied any suicidal or homicidal ideation. The patient admitted using illicit drugs in the past including marijuana, Ecstasy, Molly, and alcohol but could not quantify them. Urine toxicology testing was performed along with urinary cannabinoid and 3,4-methylenedioxymethamphetamine (Ecstasy) testing; all test results were negative. Assays for thyroid-stimulating hormone, the venereal disease research laboratory test for syphilis, antinuclear antibody, and an enzyme-linked immunosorbent assay for the human immunodeficiency virus were also ordered as a part of her workup; all results were within normal limits. A complete metabolic panel was ordered, and her serum calcium was found to be low (4.14 mg/dL). An electrocardiogram and echocardiogram were performed, but their findings were normal. After an endocrinology consultation, the patient was started on intravenous calcium carbonate. She was medicated with olanzapine and aripiprazole. She was initially started on 2 mg olanzapine then gradually titrated up to 20 mg of olanzapine and 10 mg of aripiprazole, but these displayed marked extrapyramidal side effects, specifically increased rigidity and dystonia of the neck and jaw muscles, so these were abruptly discontinued. Given her poor response to multiple antipsychotics, exaggerated extrapyramidal symptoms (EPS), reaction to olanzapine, and suboptimal absolute neutrophil count, she was started on low-dose quetiapine at 25 mg and gradually titrated up to 400 mg twice a day (BID) in the hospital to target her psychosis, lorazepam to address her agitation, and benztropine for her EPS. We also considered using a newer agent, pimavanserin to target her psychosis given the rapid development of Parkinsonian features after the use of olanzapine, but this could not be initiated due to the patient’s insurance’s reluctance to cover it. The patient showed improvement of her psychosis on quetiapine 400 mg BID, becoming more logical and goal-oriented.

A computed tomography (CT) scan without contrast of the head was ordered as a workup of her first-time psychosis. The CT showed extensive bilateral asymmetrical calcifications involving the bilateral basal ganglia including the globus pallidus, putamen, caudate, thalamus, dentate, and subcortical white matter.

The patient had a history of motor vehicle injury (MVI) three years ago as a pedestrian. From the MVI, she suffered facial trauma, splenic laceration, and an L3-L4 transverse process fracture. At that time, the patient recovered completely with no sequelae. However, a CT of her head performed at that time showed incidental bilateral basal ganglia calcifications. It was recommended that the patient take oral calcium and continue follow-up evaluations; she never did. Of note, the current CT was unchanged from the previous CT of her head performed three years ago.

The case was reviewed with the Neuropsychiatry department. They presumptively diagnosed the patient with Fahr’s syndrome based on the idiopathic basal ganglia calcification and the patient’s hypoparathyroidism, hypocalcemia, and hyperphosphatemia found via the blood work. At that point, there was also a slight concern for DiGeorge syndrome as there is evidence supporting a strong correlation between psychosis and DiGeorge syndrome. This warranted genetic testing for 22q chromosomal abnormalities once the patient was psychiatrically stable. Genetic testing by fluorescence in situ hybridization (FISH) was performed, confirming the diagnosis of a 22q11.2 deletion.

From the mother’s report, it was also evident as part of the review of the patient’s developmental history that the patient was born via normal vaginal delivery at term. The only obvious finding at birth was her long fingers. The pediatrician was consulted, but no other abnormality was found until the patient was two years old; at that time, she was diagnosed with a hearing impairment and delayed speech prompting the use of a hearing aid. Afterward, she received an ordinary education and graduated from high school.

The patient was discharged on a quetiapine 400 mg BID medication regimen. The patient is still a part of a partial hospitalization program at our hospital and is showing a marked advancement in her quality of life in terms of her behavior.

## Discussion

DiGeorge syndrome or velocardiofacial syndrome, also known in broader terms as chromosome 22q11.2 deletion, is an autosomal dominant neurogenetic condition that has been the focus of research for many years. It affects between one in 2,000 to 4,000 live births and is mostly due to a hemizygous three-megabase deletion detectable by FISH on the long arm of chromosome 22. The deletion includes the TBX1 gene between low-copy repeats LCR22-1 and LCR22-3 [2,3]. DiGeorge syndrome presents with a variable expression of phenotypes ranging from mild to severe, life-threatening forms depending on what parts of the body are involved and to the extent they are affected. Some symptoms may be present at birth while others may not be apparent until later childhood, making it imperative to screen every newborn at birth and every wellness visit if they show any associated medical or behavioral condition [4]. Associated medical conditions can include dysmorphic facial features, hypocalcemia, hypoparathyroidism (due to defective development of pharyngeal pouches), frequent infections, cardiac defects, hypernasal speech, cleft palate, autoimmune conditions, neurocognitive decline, intellectual disability, learning disorders, and various other behavioral and mental health issues.

A study published on 1,402 participants from age six to 68 years with 22q11.2 deletion showed various psychiatric disorders developed from childhood to adulthood, validating the fact that a 22q11.2 deletion is one of the strongest factors for developing psychosis [2]. Attention-deficit/hyperactivity disorder was most frequent in children with this deletion, and psychosis was more evident in 41% of adults over age 25 with this deletion [2]. Our case is unique as the patient presented with psychosis in adolescence. This highlights the fact that schizophrenia spectrum disorder is a clinical characteristic even in the pediatric and adolescence age group for individuals with a 22q11.2 deletion. A significant correlation between schizophrenia and a 22q11.2 deletion could be predicted if patients with schizophrenia have an elevated occurrence of 22q11.2 deletions or if more cases of schizophrenia are detected in patients with 22q11.2 deletion syndrome [3].

Research has shown the importance of catechol-O-methyltransferase (COMT) in developing psychosis in patients with DiGeorge syndrome. The COMT gene is one of the 24 genes within the deleted region and has a significant role in neurodevelopment. The haploinsufficiency of COMT leads to elevated levels of prefrontal dopamine in patients with 22q11.2 deletion, interfering with their cognitive abilities and contributing to their psychosis [5]. There is also research evidence supporting the theory that low levels of proline dehydrogenase and COMT in DiGeorge syndrome are responsible for the phenotypic expression of psychosis spectrum as there is a defect in the metabolism regulated by these enzymes leading to increased exposure to catecholamines and proline, increasing the individual’s vulnerability to psychotic expression [6,7].

It appears that there are risk factors that increase the susceptibility to psychosis in patients with DiGeorge syndrome. The 22q11.2 deletion is the most common genetic risk factor for the development of schizophrenia. Data indicate that when it manifests in children with a 22q11.2 deletion, they experience more severe psychiatric symptoms than other children with idiopathic developmental disabilities [8]. Psychotic features can present from age 11 until age 26 [9]. The mean age of onset of psychosis in DiGeorge syndrome is still debated as different studies have shown variation in the age of onset; this could be due to different ages of the samples being studied and the gradual evolution of psychosis in different patients. However, our patient displayed some discrepancy with the typical age of onset as she presented to the hospital in her adolescence with new-onset psychosis and with no prior psychiatric history. Her physical appearance and psychosis warranted further investigations leading to a genomic diagnosis of DiGeorge syndrome. Also, some data suggest a difference in the symptomatology of schizophrenia in patients with DiGeorge syndrome as compared to those without DiGeorge syndrome. Patients with DiGeorge syndrome exhibit neurobehavioral symptoms in addition to schizophrenia [9]. This was also obvious in our patient. Brain imaging showed bilateral basal ganglia calcifications, making us initially contemplate a rare condition called Fahr’s syndrome as a possible diagnosis in the context of psychosis. However, though it may appear in childhood or adolescence, the age of onset for Fahr’s syndrome is typically in the 40s or 50s. Genetic testing at that time excluded Fahr’s syndrome, an inherited neurological disorder characterized by abnormal calcium deposition in parts of the brain that control body movements. Symptoms may range from deteriorated motor function to dementia, seizures, and Parkinson’s-like disease [10]. After genetic confirmation of DiGeorge syndrome, it appeared the most likely reason for psychiatric disturbances in our patient was due to the 22q11.2 deletion concomitantly associated with the basal ganglia calcification observed. Patients suffering from DiGeorge syndrome rarely develop basal ganglia calcification, but in her case, those calcifications were seen on the CT of her head correlating with her current presentation. These basal ganglia calcifications can impart an increased risk of Parkinson’s disease in the future, apart from her current psychosis and can be sensitive to the development of EPS after the use of antipsychotics.

## Conclusions

In light of the above case report and discussion, DiGeorge syndrome serves as a hereditary model for the development of schizophrenia. It is therefore imperative that psychiatrists should evaluate every young individual with a 22q11.2 deletion for symptoms of psychosis. Given the fact that schizophrenia and a 22q11.2 deletion are strongly correlated, it is also recommended to obtain genetic testing in patients with recent onset psychosis if they show features of DiGeorge syndrome. More research should also be conducted to explore the role of alternative agents like pimavanserin that have a strong predilection for serotonin receptors rather than dopamine receptors in treating psychosis in patients with a 22q11.2 deletion. Also, more cases should support the incidence of basal ganglia calcification in patients with a 22q11.2 deletion (DiGeorge syndrome) as this is a rare finding evident in our patient.
